# To Our Friends

**Published:** 1857-07

**Authors:** 


### EDITORIAL AND MISCELLANEOUS.

### TO OUR FRIENDS.

We are earnestly laboring to build up a journal, that will not only be creditable to those connected with its management, and responsible for its publication, but one that will in some degree aid in elevating the character and position of the medical profession of our own State and of the whole southern country; to this end we would again urge our friends to a more diligent and regular contribution to our Journal.

We have many friends who are fully competent to keep our pages filled with matter that would greatly advance our profession, as well as themselves, who are doing nothing in this way—this should not be—we cannot write to them individually, but would say, through this medium, let us hear from you more frequently.

